# Deep Learning Classification of Systemic Sclerosis Skin Using the MobileNetV2 Model

**DOI:** 10.1109/OJEMB.2021.3066097

**Published:** 2021-03-17

**Authors:** Metin Akay, Yong Du, Cheryl L. Sershen, Minghua Wu, Ting Y. Chen, Shervin Assassi, Chandra Mohan, Yasemin M. Akay

**Affiliations:** Biomedical Engineering DepartmentUniversity of Houston 14743 Houston TX 77204 USA; Division of Rheumatology and Clinical Immunogenetics, Department of Internal Medicine UTHealth 570848 Houston TX 77030 USA

**Keywords:** Autoimmune, deep learning, mobilenet, SSc skin, unet

## Abstract

*Goal:* Systemic sclerosis (SSc) is a rare autoimmune, systemic disease with prominent fibrosis of skin and internal organs. Early diagnosis of the disease is crucial for designing effective therapy and management plans. Machine learning algorithms, especially deep learning, have been found to be greatly useful in biology, medicine, healthcare, and biomedical applications, in the areas of medical image processing and speech recognition. However, the need for a large training data set and the requirement for a graphics processing unit (GPU) have hindered the wide application of machine learning algorithms as a diagnostic tool in resource-constrained environments (e.g., clinics). *Methods:* In this paper, we propose a novel mobile deep learning network for the characterization of SSc skin. The proposed network architecture consists of the UNet, a dense connectivity convolutional neural network (CNN) with added classifier layers that when combined with limited training data, yields better image segmentation and more accurate classification, and a mobile training module. In addition, to improve the computational efficiency and diagnostic accuracy, the highly efficient training model called “MobileNetV2,” which is designed for mobile and embedded applications, was used to train the network. *Results:* The proposed network was implemented using a standard laptop (2.5 GHz Intel Core i7). After fine tuning, our results showed the proposed network reached 100% accuracy on the training image set, 96.8% accuracy on the validation image set, and 95.2% on the testing image set. The training time was less than 5 hours. We also analyzed the same normal vs SSc skin image sets using the CNN using the same laptop. The CNN reached 100% accuracy on the training image set, 87.7% accuracy on the validation image set, and 82.9% on the testing image set. Additionally, it took more than 14 hours to train the CNN architecture. We also utilized the MobileNetV2 model to analyze an additional dataset of images and classified them as normal, early (mid and moderate) SSc or late (severe) SSc skin images. The network reached 100% accuracy on the training image set, 97.2% on the validation set, and 94.8% on the testing image set. Using the same normal, early and late phase SSc skin images, the CNN reached 100% accuracy on the training image set, 87.7% accuracy on the validation image set, and 82.9% on the testing image set. These results indicated that the MobileNetV2 architecture is more accurate and efficient compared to the CNN to classify normal, early and late phase SSc skin images. *Conclusions:* Our preliminary study, intended to show the efficacy of the proposed network architecture, holds promise in the characterization of SSc. We believe that the proposed network architecture could easily be implemented in a clinical setting, providing a simple, inexpensive, and accurate screening tool for SSc.

## I. Introduction

Systemic sclerosis (SSc) is an autoimmune disease characterized by wide-spread fibrosis of the skin and internal organs. Based on the extent of skin involvement, the disease can be classified into two types: limited cutaneous SSc (LcSSc) and diffuse cutaneous SSc (DcSSc). Both subsets can include internal organ involvement, with more severe organ involvement occurring more frequently in DcSSc [1]. The internal organ complications of SSc are associated with high case-specific disability rates and mortality rates, posing a heavy socio-economic burden for society. Several studies have shown that organ involvement could occur far earlier than expected in the early phase of the disease [2], [3]. However, since SSc is an uncommon disease with multiple heterogeneous symptoms, early diagnosis and determining the extent of disease progression pose significant challenge for physicians, even at expert centers [4], [5], resulting in delays in therapy and management. Clinical diagnosis of SSc takes many factors into account. One fully validated, feasible method to determine skin thickness is the modified Rodnan skin thickness score (mRSS). In this assessment, the physician measures the skin thickness by manually palpating the skin at 17 sites on the patient's body and then uses a 0–3 scale to indicate the thickness [6]. The total score is the sum of the individual skin area, with a higher score indicative of severe skin thickening. However, as a subjective measurement, mRSS heavily depends on the physicians' palpation experience [7]–[9], and it is also not sensitive to changes over short-time periods [10]. Furthermore, many uncontrollable factors could bias the palpation test, such as the early edematous stages of the disease [11] and the impact of menopause on skin thickness [12]. All of these factors would result in marked inter-and intra-observer variabilities in detecting and monitoring SSc skin involvement. Therefore, there is an unmet need for a more consistent, reliable and objective diagnostic approach for SSc.

The sudden explosion of voluminous and complex data sets in medicine, biology, and engineering has created important challenges in 1) accurately analyzing, interpreting, and modeling unstructured data sets, and 2) converting unstructured data sets into a deeper understanding of complex, inter-related phenomena. Machine learning techniques have been developed and widely used to understand large data sets in medical image processing, speech recognition, computer vision, language processing, bioinformatics, and drug design [13], [14]. Previous studies have investigated machine learning in bioinformatics [15], biology and medicine [16], computational biology [17], [18], biomedicine [19], [20], and super resolution imaging [21]. Statistical and machine learning techniques have also been used in psychiatry [22], [23]. It has also shown potential in autoimmune disease diagnosis, prognosis prediction, and classification of diseases such as lupus [24], [25], rheumatoid arthritis [26], [27], and inflammatory bowel disease [28]. Machine learning techniques, especially deep neural networks (DNNs), have been effectively used in several biomedical applications, including protein structure prediction [29]–[31], anomaly classification [32]–[34], segmentation [35], recognition [36], [37], and brain decoding [38] since DNNs can infer suitable high-level representations without much domain-specific knowledge and prior feature construction. Also, recent advances in pre-training and transfer-training procedures have enabled DNNs to navigate complex optimization landscapes more efficiently [39]–[41].

Among several deep learning networks, Convolutional Neural Networks (CNNs) are the most commonly used in engineering, medicine, and biology. In conventional DNNs, efficient feature representations of the input space are iteratively constructed through a set of auto-encoding stages. Their success in biomedical applications has been limited due to the size of the available training sets, the size of the considered networks, and computational challenges. To overcome these difficulties with CNNs, the UNet, a modified CNN architecture with fully dense connectivity (FD)-UNet was proposed [42]. The performances of the FD-UNet and CNNs for removing artifacts from 2D Photoacoustic Tomography images generated from synthetic phantoms (circles, Shepp-Logan, and vasculature) and an anatomically realistic mouse brain vasculature dataset were compared [43]. The results showed that the FD-UNet proved to be superior and more compact than CNNs for removing artifacts and improving image quality.

In this paper, we propose a novel network architecture based on the UNet, a dense connectivity CNN, with newly added classifier layers, which works with limited training data to yield better image segmentation and more accurate classification than traditional methods. In addition, the computational efficiency and diagnostic accuracy of the network were improved by training the network with the MicroNetV2 model [44], to assess and classify SSc.

## II. Materials and Methods

### A. Deep Neural Networks

An artificial neural network (ANN) is a computational platform composed of inter-connected nodes (‘artificial neurons’) that resemble and mimic the brain's neuronal functions. The connections between the nodes, or edges, strengthen or weaken as the learning process progresses. A traditional ANN contains an input layer (senses and detects signals within the environment), a hidden layer (processes the signals sent by the input layer), and an output layer (the response to a signal or stimulus).

To improve the performance of the ANNs, two learning algorithms have been developed using statistics and analytics.  The process involves inputting data into the ANN without being given specific instructions.  Supervised learning feeds categorized data into the input layer, and the output layer returns the category with the highest score.  Unsupervised learning is like supervised learning but feeds uncategorized data into the input layer.  These conventional algorithms for machine learning require human expertise to identify the appropriate features for the ANN to perform certain tasks.

A DNN uses the same architecture of layered, interconnected nodes as an ANN but consists of more hidden layers between the input and output layers.  Within the hidden layers, each input vector is comprised of the previous layer's output vector to produce a weighted sum, which results in sequentially computed output values.  This process can represent more abstract data effectively by ignoring irrelevant information and attending to small details.  In CNN, the most common DNN, auto-encoding stages iteratively construct feature representations of the input domain.  Due to the limited size of available training sets, the large size of the required layers, and time consuming computational graphical processing units (GPU), success of using Computational DNNs in biomedical applications has been limited.

### B. Proposed Neural Network Architecture

To overcome these challenges with CNNs, in this study we propose a more elegant deep learning architecture designed to work with very few training images and yield more precise classifications. The main idea is to supplement a usual contracting network by successive layers, where pooling operators are replaced by up sampling operators. Hence, these layers increase the resolution of the output [42], [43].

In order to localize, high resolution features from the contracting path are combined with the up sampled output. A successive convolution layer can then learn to assemble a more precise output based on this information. One important modification in our architecture is that we also have a large number of feature channels in the up-sampling section, which allow the network to propagate context information to higher resolution layers. The input images and their corresponding segmentation maps are used to train the network with the stochastic gradient descent implementation of Caffe [42].

### C. MobileNetV2 Stage

**Fig. 1.**
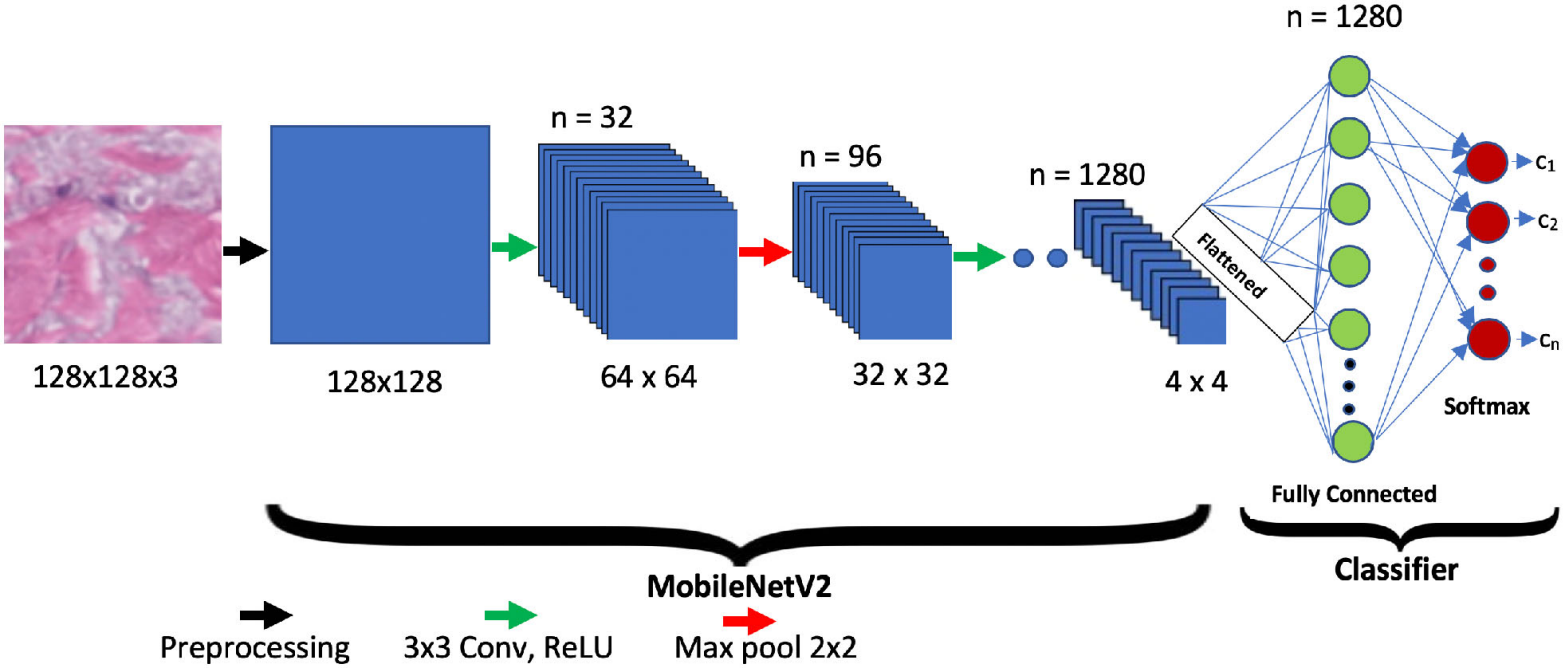
The proposed MobileNetV2 network architecture.

The proposed network architecture, shown in Fig. 1 for multi-classification, consists of a contracting path (left side) and a classifier head (right side). The contracting path follows the typical architecture of a convolutional network by using the repeated application of two 3 × 3 convolutions (unpadded convolutions), each followed by a rectified linear unit (ReLU) and a 2 × 2 max pooling operation with stride 2 for down sampling. These three processing stages, called a “block,” are repeated multiple times (making the network deep), resulting in a set of fully connected layers (classifier stage). The convolution layers obtain local weighted sums (named ‘feature maps’) at every layer by computing filters that are repeatedly applied across the entire data set to improve training efficiency. The non-linear layers then increase the feature maps’ non-linear properties. Finally, the pooling layer performs sub-sampling of non-overlapping regions in feature maps to enable the network to aggregate local features to identify complex features. At each down sampling step, we double the number of feature channels. Max pooling takes the largest element from the rectified feature map. Taking the largest element could also take the average pooling.

### D. Classifier (Fully Connected Layers) Stage

At the end of the last block of the MobileNetV2 stage, the feature map matrix is flattened into vector form and fed into a fully connected layer, like a neural network, called the classifier stage. With the fully connected layers, we combined these features together to create a model.

Finally, we have an activation function, Softmax, to classify the outputs as a SSc or a normal skin sample. Then, in an additional study, we classified the skin sample images as normal, early (mild/moderate) SSc, or late stage SSc.

### E. Preprocessing Skin Images

Skin images were collected from 20 normal subjects and 20 subjects with early (mild/moderate) and late (severe) stage SSc from the University of Texas Health Science Center, Houston, TX (UTHSC). The study was approved by the Institutional Review Board of UTHSC (HSC-MS-06-0063). The raw skin images were 512 × 512 pixels in svs format. The image size was reduced to 128 × 128 pixels in jpg format using Image J software. Then, these images were further preprocessed using image augmentation (flipping, cropping, enlarging) and standard scaling to make all the pixel values to lie between [−1, 1].

### F. Training with a Pre-Trained Model

In deep networks with many convolutional layers and different paths through the network, a good initialization of the weights is extremely important. Otherwise, parts of the network might give excessive activation, while other parts may never contribute. Ideally, the initial weights should be adapted such that each feature map in the network has approximate unit variance.

Since we work with a small dataset in biomedical applications, it is a common practice to take advantage of features learned by a model trained on a larger dataset in the same domain. This is done by instantiating the pre-trained model and adding a fully connected classifier on top. A pre-trained model is a saved network that was previously trained on a large dataset, typically on a large-scale image-classification task. The pre-trained model is "frozen," and only the weights of the classifier are updated during training. In this case, the convolutional base extracted all the features associated with each image, and a classifier was trained to determine the image class given that set of extracted features.

In this study, we used all the model features extracted from the MobileNetV2 model pretrained on the ImageNet dataset with 1.4M images and 1000 classes for all the training parameters in the MobileNetV2 stage of our network architecture [44]–[46].

### G. Fine-Tuning a Pre-Trained Model

One way to increase performance even further is to train (or "fine-tune") the weights of a few selected layers of the pre-trained model alongside the training of the added classifier. The training process will force the weights to be tuned from generic feature maps to features associated specifically with the dataset.

In most convolutional networks, the higher up a layer is, the more specialized it is. The first few layers learn very simple and generic features that generalize to almost all types of images. As you go higher up, the features are increasingly more specific to the dataset on which the model was trained. The goal of fine-tuning is to adapt these specialized features to work with the new dataset rather than overwrite the generic learning.

## III. Results

### A. Study 1: Classification of SSc

**Fig. 2.**
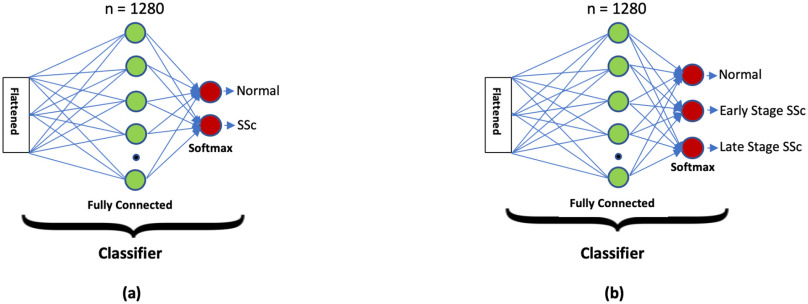
The proposed 2-class and 3-class classifiers for the MobileNetV2 architecture.

**Fig. 3.**
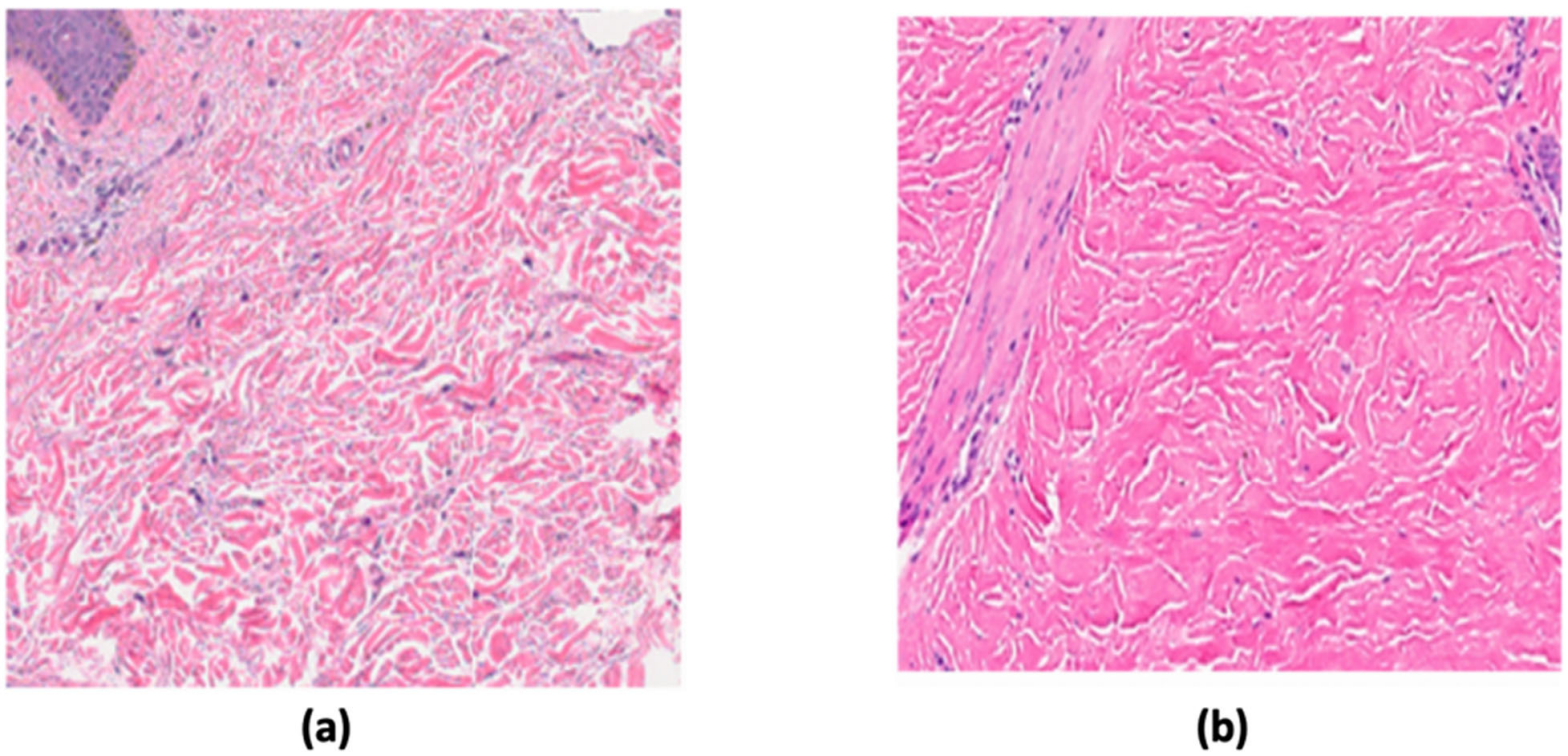
Representative normal skin image (a) and SSc (b).

In this study, a total of 1888 (1042 normal and 846 SSc) post-augmentation images were used for training, validation, and testing studies. Please note that the SSc group included both early and late stage SSc and the network classifier was modified to classify normal or SSc skin images as shown in Fig. 2(a). 80% of the total images (834 normal and 678 SSc) images were used in the training phase. Then, ∼10% of them (104 normal and 84 SSc images) were used in the validation study. Finally, the remaining ∼10% (104 normal and 84 SSc) were used in the testing study. Fig. 3 shows representative normal and SSc 128 × 128 pixel skin images used in this study.

Transfer learning from the pre-trained network model was performed [44]–[46]. Initially, for the first 100 epochs, the pre-trained weights from the MobileNetV2 were used as the fixed weights of the MobileNetV2 stage of our network architecture, consisting of 53 layers in 17 blocks with 2259265 parameters. However, the weights of the fully connected layers in the classifier head, consisting of 4 layers and 1280 parameters, were trained for a dense SoftMax layer. The network was implemented in a laptop (2.5 GHz Intel Core i7).

After 100 epochs, we maintained the weights (34112 parameters) of the first 10 layers. However, we fine-tuned the weights (2225153 parameters) of the remaining layers (from 11^th^ to 53^rd^ layers) with the weights (1280 parameters) of the fully connected layers in the classifier stage. The training time was less than 5 hours over 150 epochs.

**Fig. 4.**
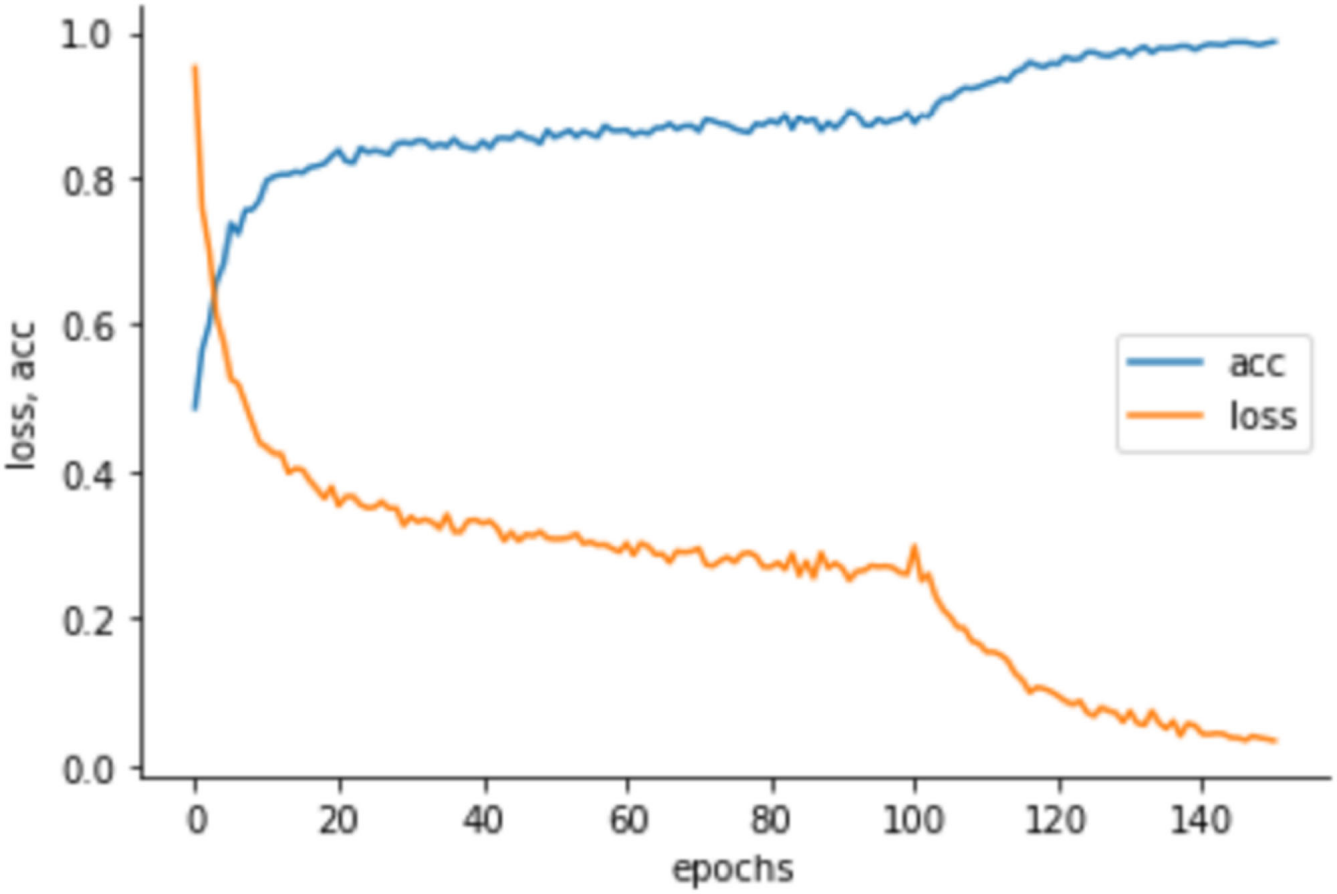
Learning curves of the MobileNetV2 training accuracy (acc)/loss for the 2-class classifier. Note the network was finetuned after the 100^th^ epoch.

Fig. 4 shows the learning curves of the training accuracy/loss after fine-tuning. We estimated the training loss, the sum of errors made for each epoch in the training set, to determine how well our model is performing during training after each iteration of optimization. We also estimated the training accuracy, estimated after the calculation of model parameters in percentage, to determine the accuracy of our model compared to the true data.

For the training phase, our networks correctly diagnosed 1512 of 1512 SSc image sets and none was misdiagnosed, yielding a sensitivity of 100%. The overall accuracy of the model was 100%.

For the validation phase, our networks correctly diagnosed 81 of 84 SSc image sets and misdiagnosed 3 of them, yielding a sensitivity of 96.4%. However, 101 out of 104 normal image sets were correctly classified and 3 of them were misclassified, yielding a specificity of 97.1% and an overall accuracy of 96.8%.

For the testing phase, our networks correctly diagnosed 80 of 84 SSc image sets and misdiagnosed 4 of them, yielding a sensitivity of 95.2%. However, 99 out of 104 normal image sets were correctly classified and 5 of them were misclassified, yielding a specificity of 95.1% and an overall accuracy of 95.2%.

**Fig. 5.**
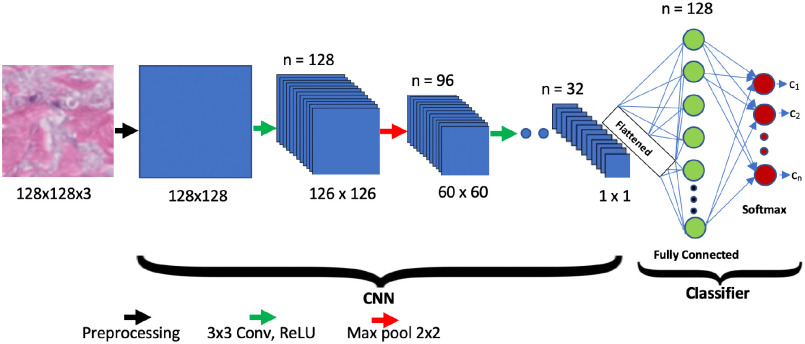
The convolutional neural network (CNN) architecture.

**Fig. 6.**
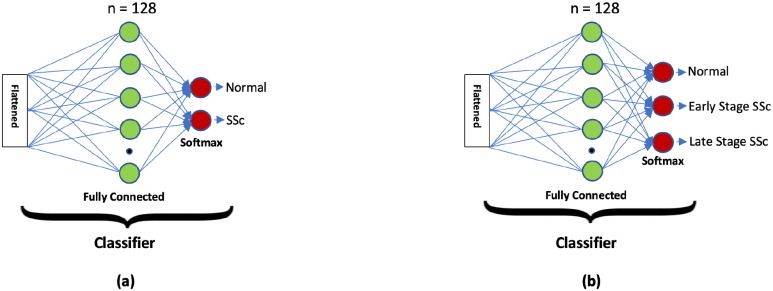
The CNN 2-class and 3-class classifier architectures.

**Fig. 7.**
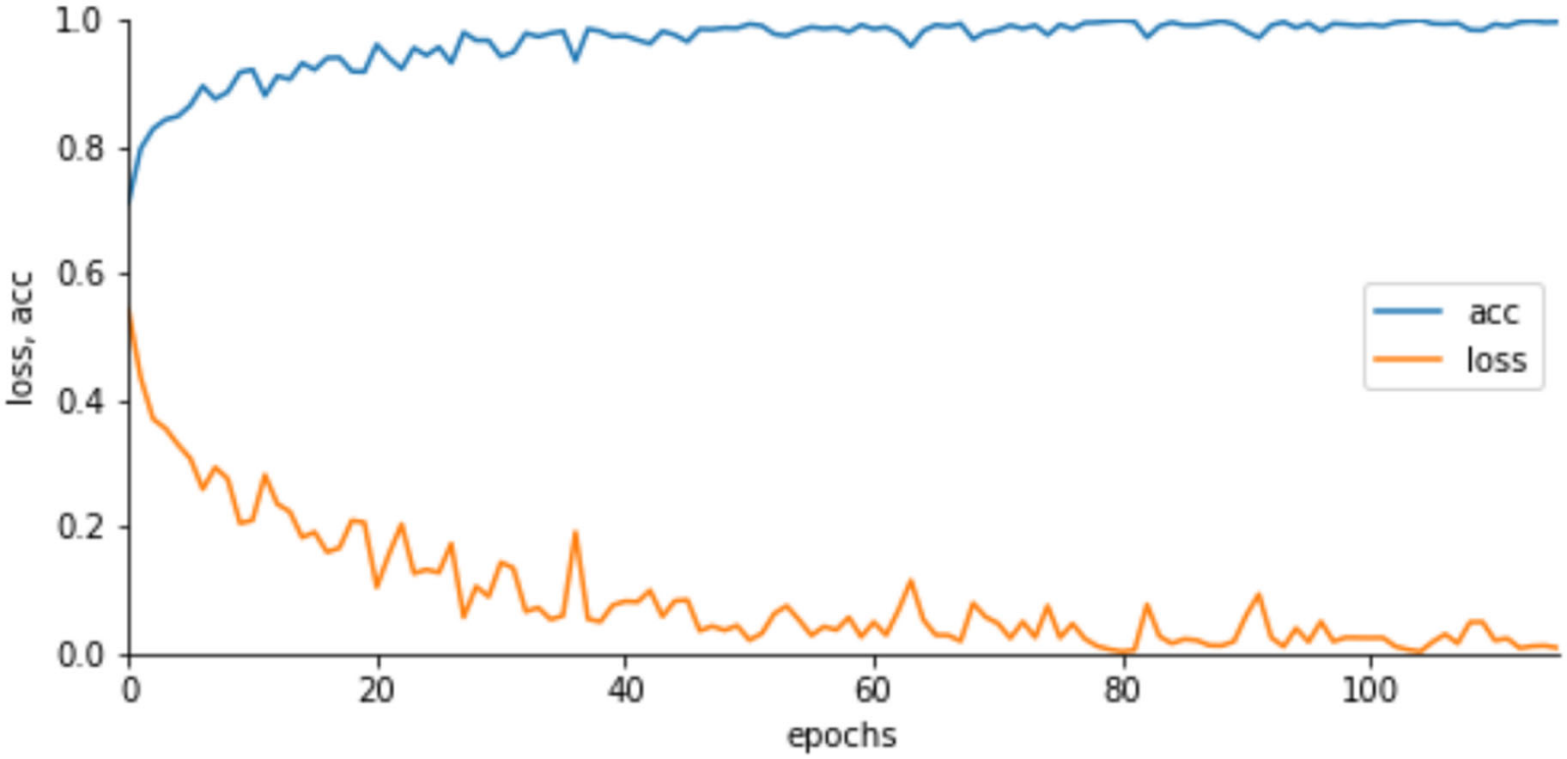
Learning curves of the CNN training accuracy (acc)/loss for the 2-class classifier.

We also analyzed the same normal vs SSc skin image sets using the CNN as shown in Fig. 5 and the same laptop. Although this method reached 100% accuracy on the training image set, it took more than 14 hours to train it over 120 epochs. The network classifier was modified to classify normal or SSc skin images as shown in Fig. 6(a). Fig. 7 shows the learning curves of the training accuracy/loss for the CNN architecture.

For the validation phase, the CNN correctly diagnosed 65 of 84 SSc image sets and misdiagnosed 19 of them, yielding a sensitivity of 77.3%. However, 100 out of 104 normal image sets were correctly classified and 4 of them were misclassified, yielding a specificity of 96.1% and an overall accuracy of 87.7%.

For the testing phase, the CNN correctly diagnosed 60 of 84 SSc image sets and misdiagnosed 24 of them, yielding a sensitivity of 71.4%. However, 96 out of 104 normal image sets were correctly classified and 8 of them were misclassified, yielding a specificity of 92.3% and an overall accuracy of 82.9%.

These results indicated that the MobileNetV2 architecture is more accurate and efficient compared to the CNN to classify normal and SSc skin images.

### B. Study 2: Classification of Early (Mild and Moderate) and Late (Severe) Stage SSc

**Fig. 8.**
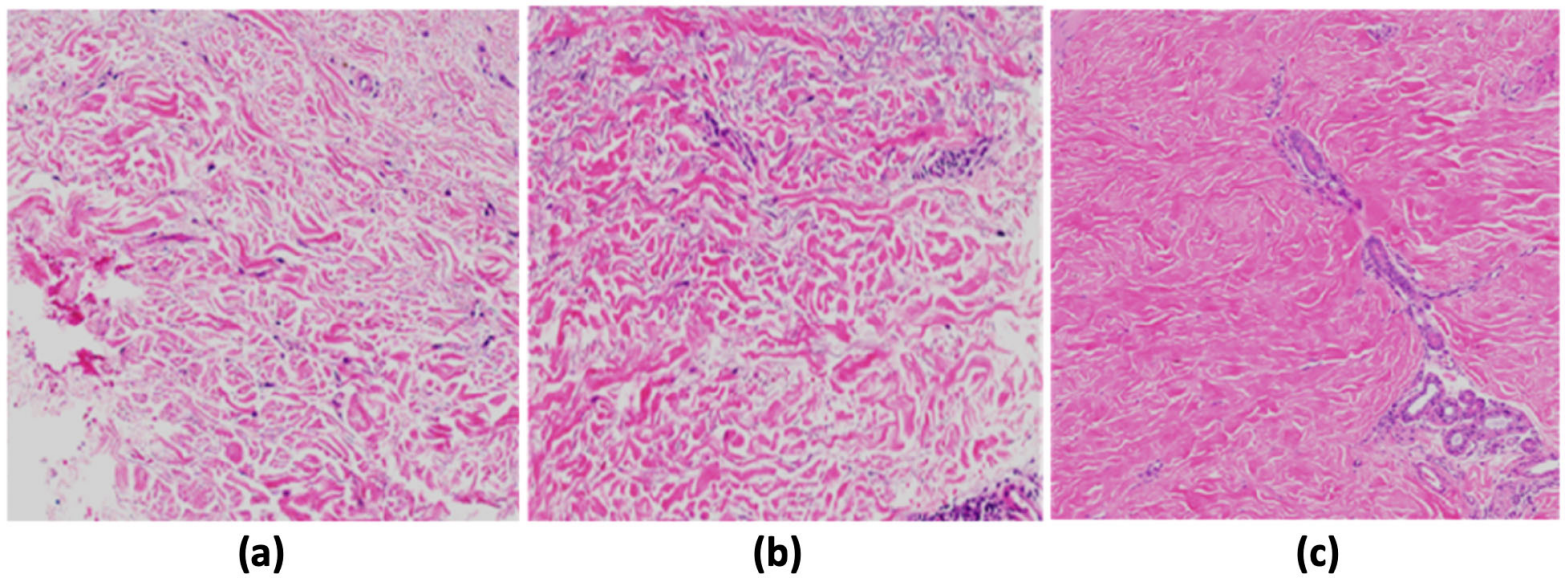
Representative normal skin image (a), early (mild/moderate) stage SSc (b) and late (severe) stage SSc (c).

In this study, an additional 1887 (1041 normal, 423 early and 423 late stage SSc) post-augmentation images were used for training, validation, and testing studies to classify both early and late stage SSc using the MobileNetV2 architecture as shown in Fig. 1. The network classifier was modified to classify normal or early or late stage SSc as shown in Fig. 2(b). 80% of the total images (834 normal. 334 early and 344 late stage SSc) images were used in the training phase. Then, ∼10% of them (103 normal, 40 early and 40 late stage SSc images) were used in the validation study. Finally, the remaining ∼10% (104 normal, 49 early and 39 late stage SSc) images were used in the testing study. Fig. 8 shows representative normal, early stage and late stage SSc 128 × 128 pixel skin images in this study.

Transfer learning from the pre-trained network model was performed. Initially, for the first 200 epochs, the pre-trained weights from the MobileNetV2 were used as the fixed weights of the MobileNetV2 stage of our network architecture, consisting of 53 layers in 17 blocks with 2263108 parameters. However, the weights of the fully connected layers in the classifier head, consisting of 4 layers and 1280 parameters, were trained for a dense Softmax layer. The network was implemented in a laptop (2.5 GHz Intel Core i7).

After 200 epochs, we maintained the weights (37664 parameters) of the first 10 layers. However, we fine-tuned the weights (2225444 parameters) of the remaining layers (from 11^th^ to 53^rd^ layers) with the weights (1280 parameters) of the fully connected layers in the classifier stage.

For the training phase, our networks correctly diagnosed 1512 of 1512 SSc image sets and none was misdiagnosed, yielding an overall accuracy of the model at 100%.

For the validation phase, our networks correctly diagnosed 178 of 183 normal, early and late stage SSc image sets. 1 out of 103 normal image sets was misclassified as early stage SSc, 2 out of 40 early stage were misclassified as normal and 2 out of 40 early stage were misclassified as late stage. Finally, all 40 late stage SSc were correctly diagnosed, yielding an overall accuracy of 97.2%.

For the testing phase, our networks correctly diagnosed 182 of 192 normal, early and late stage SSc image sets. 1 out of 104 normal image sets was misclassified as early stage SSc, 2 out of 49 early stage were misclassified as normal and 6 out of 49 early stage were misclassified as late stage, 1 out of 39 late stage was misclassified as early stage, yielding an overall accuracy of 94.8%.

We also analyzed the same normal, the early and late SSc skin image sets using the CNN as shown in Fig. 5 and the same laptop. Although this method reached 100% accuracy on the training image set, it took more than 14 hours to train it over 120 epochs. The network classifier was modified to classify normal, the early and late SSc skin images as shown in Fig. 6(b).

For the validation phase, our networks correctly diagnosed 160 of 183 normal, early and late stage SSc image sets. 9 out of 103 normal image sets were misclassified as early stage SSc, 7 out of 40 early stage SSc were misclassified as normal and 2 out of 40 early stage SSc were misclassified as late stage, 3 out of 40 late stage SSc were misclassified as normal and 2 out of 40 late stage SSc were misclassified as early stage, yielding an overall accuracy of 87.4%.

For the testing phase, our networks correctly diagnosed 168 of 192 normal, early and late stage SSc image sets. 4 out of 104 normal image sets were misclassified as early stage SSc and 1 out of 104 normal was misclassified as late stage SSc, 10 out of 49 early stage SSc were misclassified as normal and 4 out of 49 early stage SSc were misclassified as late stage SSc, 1 out of 39 late stage SSc was misclassified as normal and 4 out of 39 late stage SSc were misclassified as early stage SSc, yielding an overall accuracy of 87.5%.

These results indicated that the MobileNetV2 architecture is more accurate and efficient compared to the CNN to classify normal, the early and late SSc skin images.

## IV. Discussion and Conclusion

The successful applications of DNN in image classification and segmentation, speech recognition, and biomedical and healthcare research [29]–[41] encouraged us to use deep learning in the classification of SSc. However, several medical applications have been hampered due to the limited medical data size for the training and the requirement of the use of a GPU supercomputer, which is a network group of computers with multiple GPUs working in tandem on a single task that enables faster processing.

To overcome these two challenges, we proposed a novel deep learning network architecture, consisting of MobileNetV2 and the fully connected layers (classifier), and implemented it in a Laptop (2.5 GHz Intel Core i7). We used the pretrained MobilNetV2 base model to train the parameters of the network for the first 100 epochs. Then, to further improve the accuracy and minimize the loss, we unfroze several selected top layers of a frozen model base and jointly trained both the newly added classifier layers and these selected layers of the base model after the first 100 epochs. This allowed us to "fine-tune" the higher-order feature representations in the base model in order to make them more relevant for the classification of SSc images. The overall process took less than 5 hours to train the network and the fine-tuning it. Our preliminary study suggests that the network architecture was capable of discriminating normal skin images from SSc skin images using a laptop with a central processing unit (CPU).

We further investigated the efficacy of the MobileNetV2 architecture to assess the severity of SSc skin into early/mid or late stages of SSc. Our preliminary study suggests that the network architecture was capable of discriminating both early and late SSc images. Our proposed network architecture performed better than the CNN on the same image sets using the same laptop.

Our ultimate goal is to use this approach as a rapid and reliable method to assess the severity of SSc using images to help dermatopathologists. Once SSc is considered as a possible diagnosis by the attending physician, this would be followed by a punch biopsy of the affected skin. Once this biopsy is processed and imaged, the proposed network architecture could assign a high-accuracy diagnosis within minutes. This saves substantial time and money, compared to how diagnoses are currently made. We plan to increase our database with more SSc images during the early, mid, and later stages of SSc. Once the performance of the network has been optimized, the system could easily be implemented in a clinical setting, providing a simple, inexpensive, and accurate diagnostic tool for SSc in the future.
